# Nutrition therapy in the era of automated insulin delivery

**DOI:** 10.3389/fendo.2025.1690486

**Published:** 2025-10-31

**Authors:** Meryem K. Talbo, Franziska K. Bishop, Daria Igudesman, Karen D. Corbin, Angelica Cristello Sarteau, Elizabeth J. Mayer-Davis, David M. Maahs

**Affiliations:** ^1^ Department of Pediatrics, Division of Pediatric Endocrinology, Stanford University, Palo Alto, CA, United States; ^2^ AdventHealth Translational Research Institute, Orlando, FL, United States; ^3^ Department of Nutrition, University of North Carolina, Chapel Hill, NC, United States

**Keywords:** type 1 diabetes, nutrition therapy, dietary patterns, eating behaviors, automated insulin delivery, adjunct-to-insulin therapy

## Introduction

Type 1 diabetes (T1D) is caused by the autoimmune destruction of pancreatic β−cells, creating a lifelong need for exogenous insulin and glucose monitoring (1). Despite therapeutic advances in T1D, cardiovascular disease (CVD) remains high in this population with up to 45% developing ≥2 CVD risk factors within ten years after diagnosis (1, 2). Additionally, CVD-related mortality remains approximately twice as high in T1D compared to the general population, even when glycemic targets are achieved (3, 4). Individualized medical nutrition therapy (MNT) is a pillar for improving glycemic outcomes, promoting adequate growth, and reducing or delaying the development of chronic complications (such as CVD) and comorbidities (such as dyslipidemia or hypertension) in persons with T1D (5). MNT delivered by a registered dietitian is associated with improved cardiometabolic markers, including a 1.0-1.9% reduction in HbA1c in people living with T1D (5). In practice, MNT for T1D management has focused on carbohydrates as the main contributor to postprandial glycemic fluctuations, but compelling evidence and practice guidelines suggest that MNT should additionally incorporate broader eating patterns, food preferences, cultural practices, relationships with food and body, culinary skills, and food security to optimize health for people living with diabetes (5–7). In this commentary, we discuss the evolution of MNT for T1D management and opportunities to revise nutritional guidelines to reflect recent therapeutic advances and improve outcomes beyond glucose management, including those related to cardiometabolic risk and quality of life.

## The road so far: the evolution of MNT for T1D management

**Figure 1:**
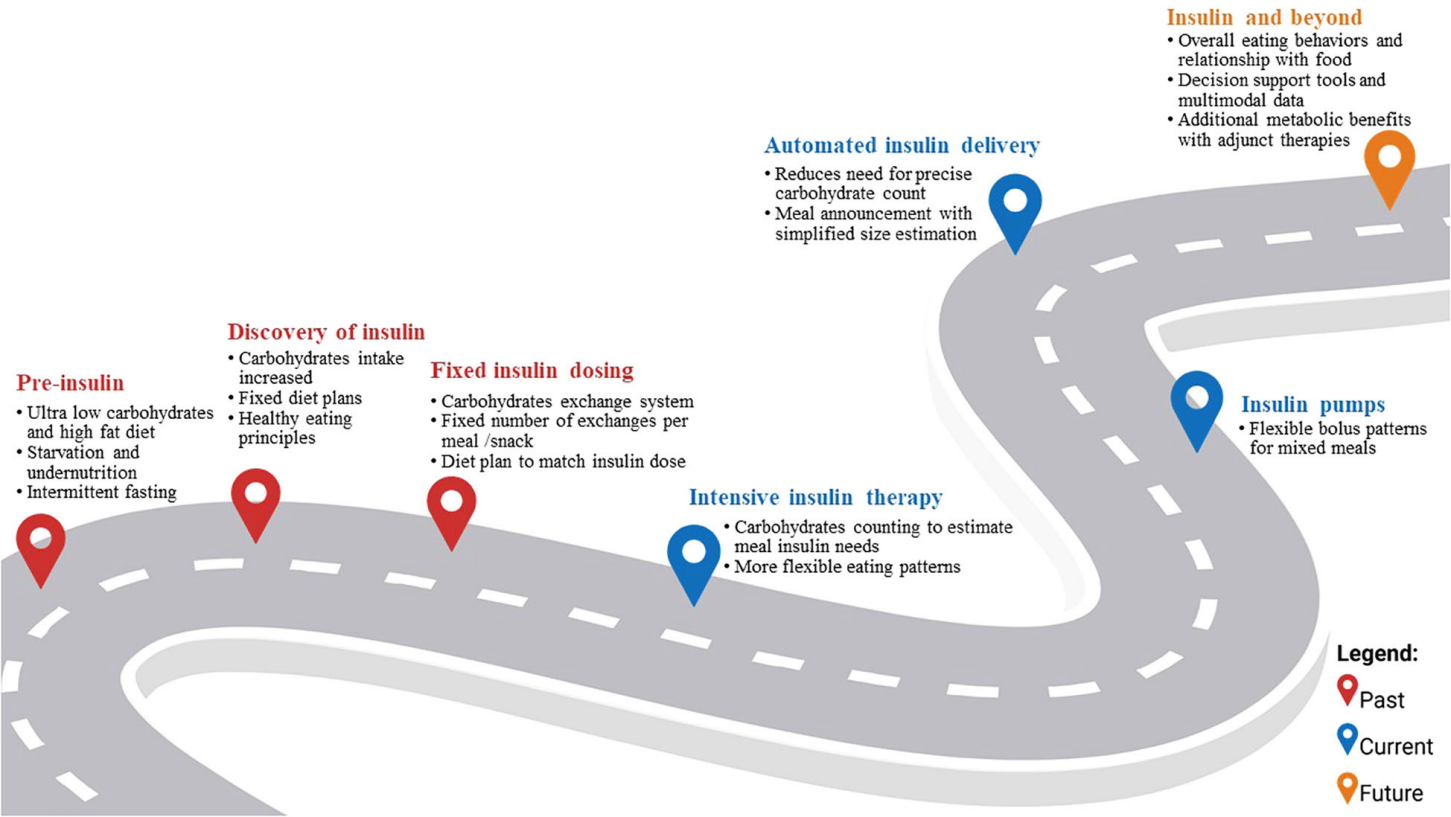
The road so far and beyond: medical nutrition therapy in type 1 diabetes.

Although MNT has transformed over the past century, it has always been a cornerstone of T1D management (**Figure 1**). Before the discovery of insulin, life expectancy after T1D diagnosis was estimated to be less than three years (8). MNT primarily took the form of carbohydrate restriction (as low as 10g of carbohydrates per day or ≤10% of daily caloric intake) to delay mortality associated with T1D (9). In some cases, high fat diets (i.e., up to 80% caloric intake from fats) and intermittent fasting regimens (i.e., once a week complete fast with just water or bouillon) were also recommended to manage glucosuria and prolong life expectancy (10, 11). Other more drastic measures like prolonged fasting (up to ten days) and hypocaloric diets were effective in reducing ketoacidosis and glucosuria but were not sustainable as they resulted in significant nutrient deficiencies, starvation, and eventual death (10, 12).

With the discovery of insulin in the early 1920s, recommended carbohydrate intake was increased to 40 to 70% of daily intake (9). Specifically, recommendations for people with T1D were to follow fixed diet plans to match carbohydrate intake to insulin doses administered (13). By the mid-20th century, and as insulin formulations and glucose monitoring developed to better reflect physiological responses to nutrient intake, a carbohydrate exchange system (1 exchange = 10 to 16 grams of carbohydrates) was proposed (14). People with T1D were prescribed a fixed number of insulin units which dictated the number and distribution of recommended carbohydrate exchanges to consume to approach euglycemia. The exchange system allowed people with T1D to choose from a variety of carbohydrate sources while matching carbohydrate amounts to their prescribed insulin dosing (15).

The development of intensive insulin therapy (i.e., three or more daily injections of insulin) and capillary blood glucose meters transformed T1D management (15). People with T1D could monitor their glycemia at home and match their insulin dose to their carbohydrate intake instead of having to rigidly adhere to predetermined carbohydrate amounts based on a fixed insulin dose. Before this, glucose monitoring relied on urine tests as blood glucose monitoring strips only became available in 1965 for use in the clinic and 1980s for at home use (16). This advancement provided added flexibility in what, when, and how to eat (15). Specifically, in the 1990s carbohydrate counting (CC, i.e., counting the number of carbohydrates found in food and using this value to estimate prandial insulin needs) became a topic of interest in the US, solidified with the Diabetes Control and Complications Trial results (15).

Although CC has remained a mainstay of the dietary management of T1D, its efficacy is equivocal, and unintended effects on diabetes distress and disordered eating are of concern (17). While some studies showed improvement in HbA1c values (standard mean difference [95%CI]: -0.51 [-0.83, -019] %) when insulin dosing was combined with precise CC and was compared to standard diabetes education (16), evidence for CC superiority is less clear when compared to other dietary approaches of matching insulin to food intake (-0.31 [-0.99, 1.61]%) such as glycemic index or fixed carbohydrate amounts (18). Discrepancies between insulin doses estimated using CC and postprandial glycemic responses can be accounted for by both dietary and non-dietary factors including fiber and other macronutrients, previous physical activity, and time of day (7, 19–21). CC is complex, takes time, requires nutritional literacy and numerical skills, and is generally inaccurate, especially when estimating carbohydrate intake from fresh and non-prepackaged foods which are the basis of a healthy diet (19, 22–24). Additionally, parallel to the general population (25), low diet quality remains a persistent challenge for people with T1D and has implications for both glycemic outcomes and downstream cardiometabolic risk, thus supporting the need to shift from a carbohydrate-centric approach to target overall diet quality (26, 27). Current national and international guidelines state that there is no one ideal diet approach for MNT in T1D; however, dietary patterns that are rich in sources of fiber and unsaturated fatty acids such as whole grains, vegetables, and lean protein, and low in added sugars and sodium, have consistently been associated with improved cardiometabolic health outcomes, including glycemia (5, 7, 28, 29). Specifically, dietary patterns rich in fiber (daily average 35 grams) have been associated with lower HbA1c (mean difference [95%CI]: -0.18 [-0.30, -0.07]%), LDL cholesterol (−0.17 [−0.27, −0.08]mmol/L), triglycerides (−0.16 [−0.23, −0.09] mmol/L), and Body Mass Index (−0.36 [-0.55, −0.16]) compared to dietary patterns lower in fiber (daily average 19 grams) in persons with diabetes (30). Similar trends have been observed in youth (<18 years of age) with T1D. In a recent study with 120 youth participants with T1D, those reporting dietary patterns rich in ultra-processed foods had a 3.5 higher odds of having higher HbA1c levels (31). Meeting nutritional guidelines remains challenging for many. In a sample with 291 families of children with T1D aged 8–18 years, the average eating patterns had less than half the recommended amount of fruits, vegetables, whole grains, nuts and seeds (source of 17% of energy intake) while 48% of daily energy intake came from refined-grain products, desserts, chips, and sweetened beverages (32). These trends are especially concerning given the rising prevalence of overweight/obesity in the T1D population, with overweight and obesity affecting 35% and 20% of adults with T1D respectively, increasing the risk of insulin resistance and CVD (33).

Additionally, the psychological burden associated with T1D such as the fear of hypoglycemia and the demands of precise CC increases the risk of developing disordered eating behaviors (5, 34, 35). The demands for precise CC to match insulin dosing can also increase meal-related anxiety and disturb intuitive eating and satiety/hunger signaling (36). Specifically, behaviors such as under-bolusing or omitting insulin to lose weight are prevalent in people with T1D, with prevalence rates up to 40% in youth and 20% in adults (37). Although limited, the current evidence supports the theory of an association between disordered eating and T1D management outcomes. In a cross-sectional analysis with 151 adolescents, participants in the “at risk for disordered eating” group had lower diet quality compared to teens who were in the low-risk group (38). Disordered eating has also been linked to higher HbA1c levels, higher risk of ketoacidosis, and chronic T1D complications (5, 39). Thus, nutrition interventions for healthful eating should be mindful of the heightened disordered eating risk in T1D.

## The road ahead: proposed strategies for adapting MNT to contemporary clinical care

Automated insulin delivery (AID) is now the standard of care as per international guidelines for youth and adults with T1D (40, 41). These systems increase time in target glucose range (TIR; i.e., 70–180 mg/dL) by 10.9% [9.4, 12.4] and reduce HbA1c by 0.37% [-0.49, -0.29] compared to non-automated insulin administration modalities (42). Currently available AID systems can ease some of the burden associated with mealtime management in T1D and could potentially reduce the need for precise CC while maintaining glycemic targets. Accordingly, the iLet™ system was designed to reduce diabetes burden and thus only allows users to indicate meal type and relative size (“usual,” “more,” or “less”) but not specific carbohydrate amounts, and decreased HbA1c from 7.9% to 7.3% and improved TIR from 51% to 65% in 219 youth and adults relative to injections, non-automated insulin pumps and hybrid closed-loop (43, 44). In another study, adolescents using Medtronic’s MiniMed™ 780G AID system maintained higher TIR (80% at 12 months follow-up) when employing carbohydrate counting versus simplified meal announcements, although those using simplified announcements still achieved TIR within recommended targets (73% at 12 months) (45). In a crossover design using the CamAPS™ system, Laesser et al. found no difference in TIR between a simplified meal announcement (69.9 ± 12.4%) and CC (70.7 ± 13.0%) (p=0.48) (46). Another team of investigators found that up to 20 grams of unannounced carbohydrates could be consumed with the MiniMed™ 780G system without significant differences in postprandial glucose compared to when the 20 grams were announced (TIR: 70.8% vs. 70.3% and time above range (TAR; i.e., >180 mg/dL): 27.6% vs. 27.1%, respectively) (47). However, larger amounts of carbohydrates (≥40 grams) resulted in significantly higher glycemic excursions when meals were not announced, such that TAR was 15-20% higher compared to when an announcement was made (47). Thus, certain AID systems allow users to achieve reasonable glycemic outcomes using a less burdensome carbohydrate announcement approach, but larger carbohydrate intakes may necessitate greater user input. Additionally, current evidence supports the glycemic impact of protein and fat on postprandial glycemia thus further complicating mixed-meal management in T1D. Recent literature reviews found that both protein and fat contribute to postprandial glycemic excursions by extending their duration and amplifying their magnitude (48, 49). Current guidelines recommend meal-time insulin adjustments for mixed meals to account for fat and protein content by modifying both dose and delivery of the insulin bolus (5, 7). Thus, parallel to the dual-wave boluses used with non-automated insulin pumps that improved postprandial glycaemia in high-fat, high-protein and low-GI meals (50), the CamAPS FX system includes a setting for “slowly absorbed meals” and the DBLG1-Diabeloop system offers a “high-fat meal” option (51). Overall, while AID systems reduce the burden of precise CC, meal announcements and a general understanding of glycemic effects of macronutrients remain important for safety and effective self-management, and thus continue to be part of the MNT. The relationships between dietary composition of meals and glycemia fluctuations are complex and vary widely between and within individuals owing to a multitude of clinical and sociodemographic factors (5). These complex interactions require the integration of multi-modal personal data to provide effective eating decision support tools for AID systems (52, 53). In order to progress the development of such tools, machine learning approaches which can automatically identify complex relationships in data should be explored. One study, proposed an eating decision support model that accounts for clinical characteristics, physical activity, insulin, glycemia, and eating behavior data in order to provide adolescents with T1D with meal timing and macronutrient composition recommendations to optimize TIR (52). The investigators’ (A.CS. and E.M-D.) propose the model as a foundation to build a mobile health application or integrate with an AID system depending on the individual’s access to diabetes technology (52). However, translation of these findings to clinical practice faces a multitude of challenges spanning issues related to affordability and access, potential for sensor or pump malfunction, cybersecurity and privacy concerns, data management, standardization and interoperability across devices, regulatory constraints, pharmacokinetic and pharmacodynamic constraints of available insulin formulations, and provider skills or bias (54).

A recent review catalogued the impact of AID systems on eating behaviors, including limited evidence from four qualitative and three quantitative studies (55). The available evidence revealed that AID systems reduce eating-related stress and increase confidence around food in people with T1D. However, some users reported increasing portion sizes and the intake of energy-dense foods, suggesting that the benefits of added food flexibility, increased quality of life, and reduced mealtime burden afforded by AID may be offset by the trend to consume more discretionary foods (55). Although the authors did not report on overall changes in diet quality, these results invite speculation that AID systems lead to changes in dietary patterns (55). As AID moves towards fully closed-loop systems that no longer require CC for every meal, nutrition education can increasingly focus on diet quality, variety, eating behaviors, and individualized meal patterns aligned with healthful eating principles (56). This shift to emphasize healthy eating patterns associated with reduction in cardiometabolic risk factors is especially important given the rising rates of overweight, obesity, and insulin resistance in T1D (57).

In addition to advancements in insulin delivery, preliminary data on adjunctive therapies like glucagon-like peptide 1 receptor agonists (GLP-1 RA) for T1D management are promising (57, 58). A recent review found an average reduction in HbA1c of 0.21% [-0.26, -0.17] and significant weight loss averaging 3.78 kg [-4.39, -3.71] with the use of GLP-1 RA (59). The delayed gastric emptying effect of GLP-1 RA could particularly benefit fully closed AID systems by creating better alignment between the delayed timing of exogenous insulin action and carbohydrate absorption (58). This mechanism mirrors the advantages seen with mixed meals containing fiber, complex carbohydrates, protein and fat, which can provide AID systems more time to respond compared to high-glycemic index or refined carbohydrate meals that cause rapid glucose spikes. While GLP-1RAs remain off-label for T1D, real-world adoption is growing, often with the prescription for obesity as an additional diagnosis. The percentage of US adults with T1D prescribed GLP-1RAs increased from 0.3% in 2010 to 6.6% in 2023 (60). Emerging evidence suggests potential for complementary benefits when combining these pharmacological approaches with advanced AID technologies (58).

## Conclusion

Current nutritional recommendations should evolve in the context of recent therapeutic advancements in AID systems and adjunctive to insulin medications. As such future research is needed to design, evaluate, and implement new MNT models to meet the evolving realities of T1D management. Given AID’s ability to compensate for inaccuracies in CC, nutritional guidance should pivot from a sole focus on precise carbohydrate quantification toward practical dietary principles emphasizing overall nutritional quality. The goal is not to eliminate education around the impact of carbohydrates and other macronutrients on glycemic excursions, but to propose a more comprehensive approach that also offers cardiometabolic protection and that can be individualized, thus empowering people with T1D to make healthier food choices without added cognitive burden or stress.
